# A p38 MAPK-Mediated Alteration of COX-2/PGE_2_ Regulates Immunomodulatory Properties in Human Mesenchymal Stem Cell Aging

**DOI:** 10.1371/journal.pone.0102426

**Published:** 2014-08-04

**Authors:** Kyung-Rok Yu, Jin Young Lee, Hyung-Sik Kim, In-Sun Hong, Soon Won Choi, Yoojin Seo, Insung Kang, Jae-Jun Kim, Byung-Chul Lee, SeungHee Lee, Andreas Kurtz, Kwang-Won Seo, Kyung-Sun Kang

**Affiliations:** 1 Adult Stem Cell Research Center, College of Veterinary Medicine, Seoul National University, Seoul, South Korea; 2 Research Institute for Veterinary Medicine, College of Veterinary Medicine, Seoul National University, Seoul, Korea; 3 Institute for Stem Cell and Regenerative Medicine in Kangstem Biotech, Biotechnology Incubating Center, Seoul National University, Seoul, Korea; 4 Berlin-Brandenburg Center for Regenerative Therapies, Berlin, Germany; RWTH Aachen University Medical School, Germany

## Abstract

Because human mesenchymal stem cells (hMSC) have profound immunomodulatory effects, many attempts have been made to use hMSCs in preclinical and clinical trials. For hMSCs to be used in therapy, a large population of hMSCs must be generated by in vitro expansion. However, the immunomodulatory changes following the in vitro expansion of hMSCs have not been elucidated. In this study, we evaluated the effect of replicative senescence on the immunomodulatory ability of hMSCs in vitro and in vivo. Late-passage hMSCs showed impaired suppressive effect on mitogen-induced mononuclear cell proliferation. Strikingly, late-passage hMSCs had a significantly compromised protective effect against mouse experimental colitis, which was confirmed by gross and histologic examination. Among the anti-inflammatory cytokines, the production of prostaglandin E_2_ (PGE_2_) and the expression of its primary enzyme, cyclooxygenase-2 (COX-2), were profoundly increased by pre-stimulation with interferon gamma (IFN-γ) and tumor necrosis factor alpha (TNF-α), and this response was significantly decreased with consecutive passages. We demonstrated that the impaired phosphorylation activity of p38 MAP kinase (p38 MAPK) in late-passage hMSCs led to a compromised immunomodulatory ability through the regulation of COX-2. In conclusion, our data indicate that the immunomodulatory ability of hMSCs gradually declines with consecutive passages via a p38-mediated alteration of COX-2 and PGE_2_ levels.

## Introduction

MSCs have been isolated from almost all tissues [1], and they exhibit a fibroblastic spindle shape and can be directed to differentiate into several different cell types, such as adipocytes, chondrocytes and osteoblasts [2]. It has been reported that MSCs play critical roles in many physiological functions, such as tissue homeostasis, regeneration and wound healing [3]. Together with their broad tissue distribution and ability to locate sites of injury, the immunomodulatory properties of MSCs hold great potential for therapeutic use [4], [5]. The immunomodulatory properties of MSCs are elicited by proinflammatory cytokines, such as IFN-γ, TNF-α and IL-1, which produced during an immune response [6]. The combination of these proinflammatory cytokines provokes the production of several inducible soluble factors, specifically, transforming growth factor-β1 (TGF-β1), prostaglandin E_2_ (PGE_2_), nitric oxide (NO) and indoleamine 2, 3-dioxygenase (IDO), which in turn induce the immunosuppressive functions of MSCs [3], [5]. Interestingly, proinflammatory cytokine-stimulated murine MSCs use NO as a major mediator to exert their immunosuppressive functions, whereas the immunosuppressive functions of proinflammatory cytokine-stimulated human MSCs are executed through IDO [7], [8]. However, PGE_2_ is secreted in both murine and human MSCs upon stimulation with inflammatory cytokines. PGE_2_ induces macrophages to produce a higher level of IL-10 through the prostaglandin EP2 and EP4 receptors [9]. Furthermore, PGE_2_ shows a strong inhibitory effect on monocyte-derived dendritic cells (DC) [10], natural killer (NK) cells and T cells [11], [12]. Previous studies reported that transplantation of human MSCs into xenogeneic disease models, including mouse, rat, rabbit and dog, showed significant improvements, suggesting that human MSCs can regulate the immune/inflammatory response in vivo with their immunomodulatory property [13]. We recently demonstrated that MSCs can suppress mononuclear cell proliferation and reduce the severity of colitis in mice by producing PGE_2_ via the nucleotide-binding oligomerization domain 2 (NOD2)-receptor-interacting serine/threonine-protein kinase 2 (RIP2) pathway [14].

Cyclooxygenase (COX) enzyme plays important roles in the biosynthesis of prostaglandins from arachidonic acid. There are two COX isoforms: COX-1 is constitutively expressed in a wide range of tissues and COX-2 is an inducible enzyme that produces PGE_2_ during inflammation [15]. p38 mitogen-activated protein kinase (MAPK) is preferentially activated by inflammatory stimuli and post-transcriptionally regulates COX-2 mRNA expression [16]. Treatment of SB203580, a specific inhibitor of p38 MAPK that acts by competing with ATP for the nucleotide binding site of p38, caused a rapid disappearance of COX-2 mRNA, suggesting that p38 MAPK is involved in the transcription and stabilization of COX-2 mRNA [17].

It is important to isolate and expand MSCs in vitro for therapeutic use. Unlike pluripotent stem cells, such as embryonic stem cells, MSCs undergo replicative senescence in vitro after 20–40 rounds of cell division, which is characterized by cell enlargement, changes in morphology, DNA damage response and growth arrest [18], [19]. We and other groups have recently reported the molecular mechanisms are controlled by the hMSC aging process. During the progression of MSC senescence, the activity of histone deacetylases (HDACs), which regulates polycomb group genes (PcGs) and jumonji domain-containing 3 (JMJD3), is down-regulated [20]. ZMPSTE24, which is involved in the post-translational maturation of lamin A, is decreased during MSC senescence, leading to the accumulation of prelamin A in the nuclear envelope [21]. MSC properties, including multilineage differentiation, proliferation, homing and wound healing, gradually become compromised as MSCs undergo senescence [22]. However, the changes in immunomodulatory properties during MSC aging in the context of COX-2/PGE_2_ expression have not yet been elucidated. An understanding of the changes in the immunomodulatory properties of MSCs during the aging process is required for their use of in clinical applications. In this study, we assessed the effect that consecutive MSC passages have on the immunomodulatory ability of hMSCs and the underlying mechanisms involved in these effects.

## Results

### Characterization of hMSCs

**Figure 1:**
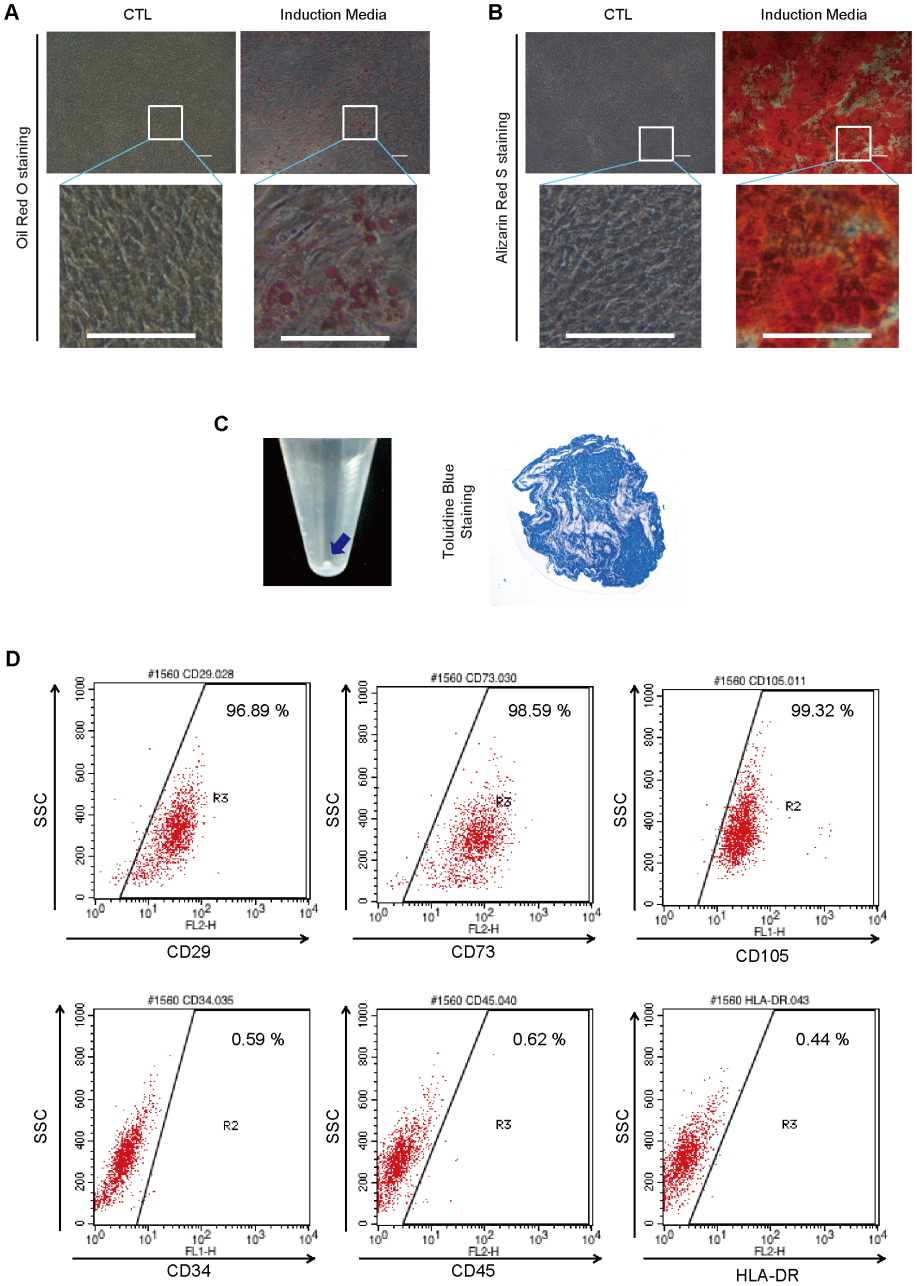
Characterization of hMSCs. (A–C) Images of differentiated hMSCs after induction into specific tissues. (A) The lipid droplet accumulation in differentiated cells was visualized using Oil Red O staining after 2 weeks of adipogenic induction. (B) Calcium deposition was stained with Alizarin Red S after 2 weeks of osteogenic induction. (C) Glycosaminoglycans in cell pellets were revealed by Toluidine blue staining after 2 weeks of chondrogenic induction. (D) hMSCs (1×10^6^ cells/ml) were stained with FITC- or PE- conjugated antibodies specific for human CD29, CD34, CD45, CD73, CD105, and HLA-DR.

To determine the differentiation capacity of hMSCs, we induced the in vitro differentiation of the adipogenic, osteogenic and chondrogenic lineages. Under adipogenic induction, accumulated lipid droplets were visualized using Oil Red O staining after 2 weeks. Mineral deposition was visualized with Alizarin Red S staining after 2 weeks of osteogenic induction. Chondrogenic differentiation was confirmed by Toluidine Blue staining after 3 weeks (Figure 1A–C). The cell surface marker expression of hMSCs was examined by flow cytometric analysis. The hMSCs were positive for stromal markers (CD29, CD73, and CD105) but negative for hematopoietic cell and leukocyte markers (CD34, CD45, and HLA-DR) (Figure 1D).

### Replicatively senescent hMSCs show senescence phenotypes with a compromised immunosuppressive ability

**Figure 2:**
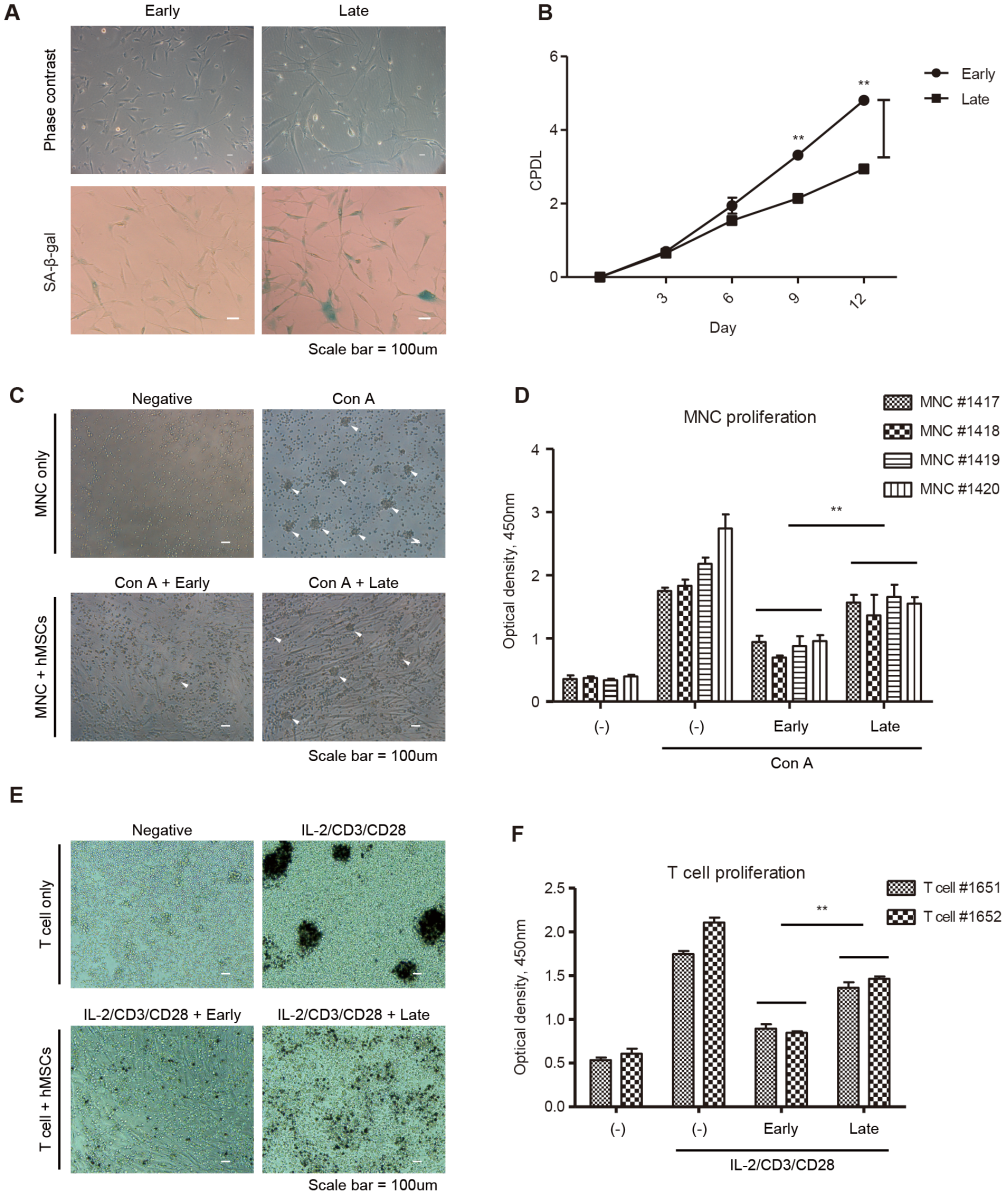
Replicative senescent hMSCs show impaired immunosuppressive ability. (A) Phase contrast images of early- and late-passage hMSCs. β-gal staining was performed in the early-passage and late-passage hMSCs to confirm their senescence. (B) The cumulative population doubling levels of hMSCs were calculated after each repeated subculture to evaluate the loss of their proliferation potential. n = 3. (**P<0.01). (C, D) hMNCs were treated with Concanavalin A to stimulate proliferation and colony formation. Stimulated hMNCs were co-cultured with early- or late-passage hMSCs at a 1∶10 ratio (MSC:MNC). White arrows indicate colonies of MNCs (C). hMNC proliferation was assessed after 72 hours using a BrdU kit (D). The results show four representative results from experiments using three different hMSC lines. Scale bar  = 100 µm. n = 3. (**P<0.01). (E, F) Human primary T cell proliferation was induced by treatment of anti-CD3 monoclonal antibody, anti-CD28 monoclonal antibody and IL-2 to hMNCs. Activated human T cells were co-cultured with early- or late-passage hMSCs at a 1∶10 ratio (MSC:MNC) (E). T cell proliferation was measured by BrdU assay after 72 hours (F). The graph shows two representative results from experiments using three different hMSC lines. Scale bar  = 100 µm. n = 3. (**P<0.01).

hMSCs must be expanded in vitro for maximum efficiency in clinical and pre-clinical applications. To determine the effects of consecutive hMSC cell divisions, we investigated the senescence phenotype and immunomodulatory properties in early-passage (5–10 passages, 10–20 population doublings) and late-passage (15–20 passages, 25–30 population doublings) hMSCs. Late-passage hMSCs exhibited a flattened shape and cell enlargement morphology as well as increased SA-β-gal activity compared to early-passage hMSCs (Figure 2A). Furthermore, late-passage hMSCs exhibited a significantly decreased proliferation rate (Figure 2B). To determine whether the immune modulatory functions of hMSCs were related to the physiological aging of the cells, we co-cultured human umbilical cord blood-derived mononuclear cells (hUCB-MNCs) with early- and late-passage hMSCs. Under cell-to-cell contact, the inhibitory effect of hMSCs on the proliferation of mitogen (Concanavalin A)-induced hUCB-MNCs was investigated. Remarkably, hUCB-MNC proliferation was suppressed more in the presence of early- passage hMSCs than late-passage hMSCs (Figure 2C, D and S1). Consistently, the decline in immunosuppressive function of late-passage hMSCs was observed when CD3/28 and IL-2 were used instead of ConA to induce the activation and proliferation of T cells specifically (Figure 2E, F). These data suggest that a decrease in the immunosuppressive properties of hMSCs correlates with the senescence induced by prolonged in vitro cell culture.

### Early- passage hMSCs show enhanced protective effects against DSS-induced colitis in mice

**Figure 3:**
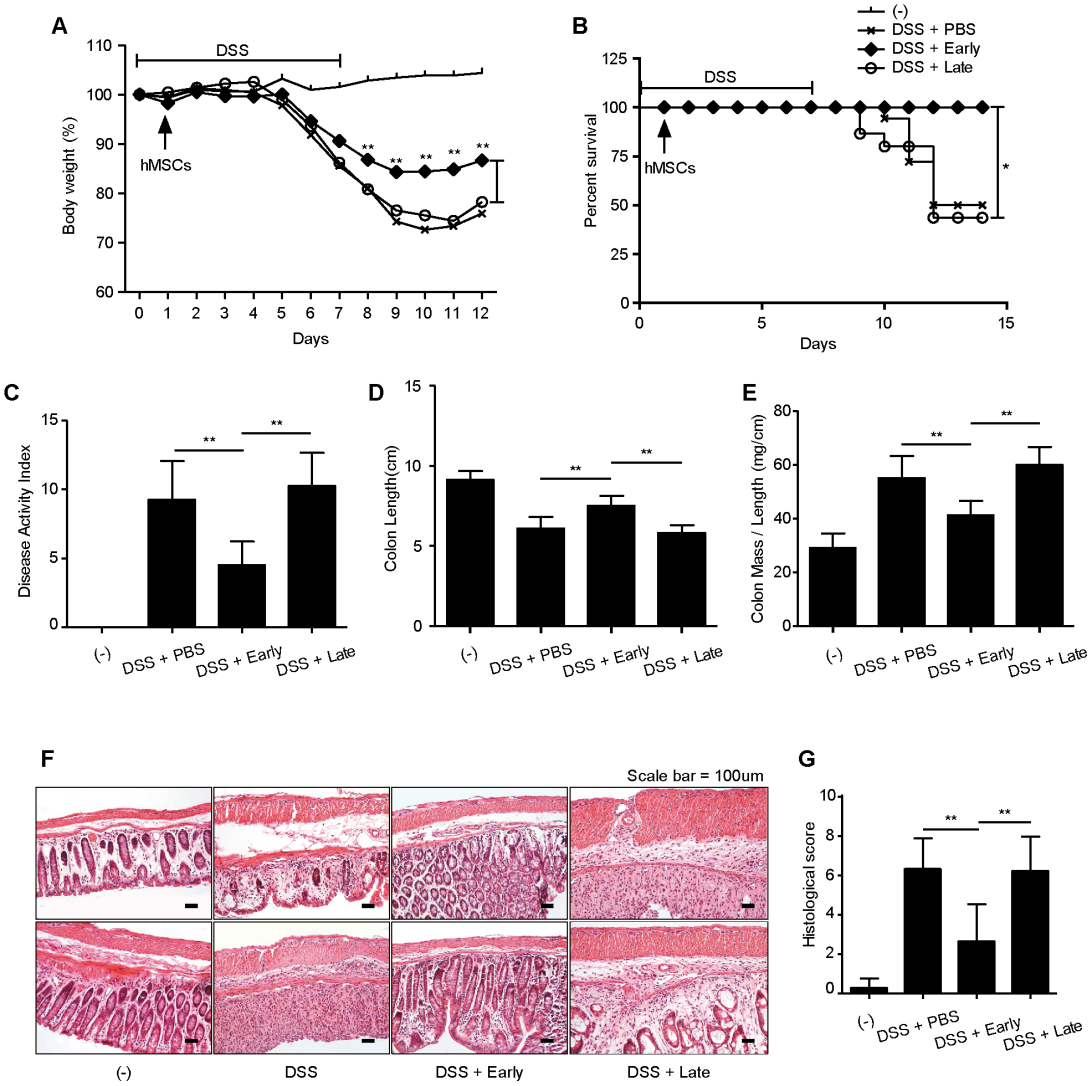
Administration of late-passage hMSCs reduces the protective effects against DSS-induced colitis in mice. (A–F) 3% DSS water was administered to mice for seven days to induce colitis. Early- or late-passage hMSCs were injected intraperitoneally one day after the administration of DSS. The percentage of body weight loss (A), the Mantel Cox analysis of survival rate (B) and the disease activity index for colitis severity (C) were monitored as clinical progression. Mice were sacrificed ten days after the induction of colitis with DSS, and the length (D), weight-to-length ratios of the colons (E) and colon sections were stained with H&E and histopathologic evaluation was investigated by determining lymphocyte infiltration and intestinal damage (F, G). (*P<0.05, **P<0.01).

To support the observation that the decrease in the hMSC immunosuppressive properties correlates with replicative senescence in vitro, we subsequently investigated the potential therapeutic efficacy of early- and late-hMSCs in the experimental model of acute colitis that was induced by oral dextran sulfate sodium (DSS) administration. It has been reported that DSS causes intestinal inflammation due to the exposure of the submucosa to exterior antigens (intestinal bacteria and food), leading to the recruitment or activation of inflammatory cells associated with innate immunity [23]. Mice were randomly assigned with similar body weight to investigate the therapeutic effect of early- and late-passage hMSC in intestinal inflammation. Oral administration of a 3% DSS solution induced acute colitis, characterized by clinical symptoms (diarrhea and bloody stool) with sustained weight loss resulting in 50% mortality (9/18). Intraperitoneal injection of early-passage hMSCs reduced the loss of body-weight and decreased the mortality of mice compared to PBS- or late-passage hMSC injections (Figure 3A and B). Strikingly, the transplantation of early-passage hMSCs rescued 100% (n = 15) of the mice from colitis-induced lethality (Figure 3B). Treatment of late-passage hMSCs, however, did not exert these beneficial effects. On day 7, the disease activity index was significantly decreased by treatment with early-passage MSCs. In contrast, the administration of late-passage hMSCs did not show beneficial effects on the disease activity index (Figure 3C). The disease activity index was measured according to standards for the quantification of symptoms of patients with Crohn's disease [24]. On day 10, the mice were sacrificed, and the entire colon from the caecum to the anus was acquired. The length, mass weight and histopathology of the colon were investigated to determine the inflammation status. The colon length decreased in mice treated with PBS or late-passage hMSCs. However, the colon length was restored by an injection of early-passage hMSCs (Figure 3D). The increased mass-to-length ratio revealed that both hyperemia and inflammation were exhibited in the colons of colitis-induced mice. Early-passage hMSCs reduced the mass-to-length ratio significantly compared to late-passage hMSCs (Figure 3E). Colon samples were processed and stained with H&E for histopathological evaluation. Histologic examination showed the destruction of the entire epithelium and submucosal edema and the infiltration of inflammatory cells into the lamina propria and submucosa in the colon of DSS-treated mice (Figure 3F). Importantly, the administration of early-passage hMSCs greatly reduced histologic damage and the histologic score in the colon. In contrast, late-passage hMSCs did not prevent histologic damage or decrease the histologic score (Figure 3F and G). These in vivo data suggest that replicative senescence significantly impairs the ability of hMSCs to deactivate the colonic inflammatory response.

### Alteration of immunomodulatory activities during hMSC aging is dependent on PGE_2_ production

**Figure 4:**
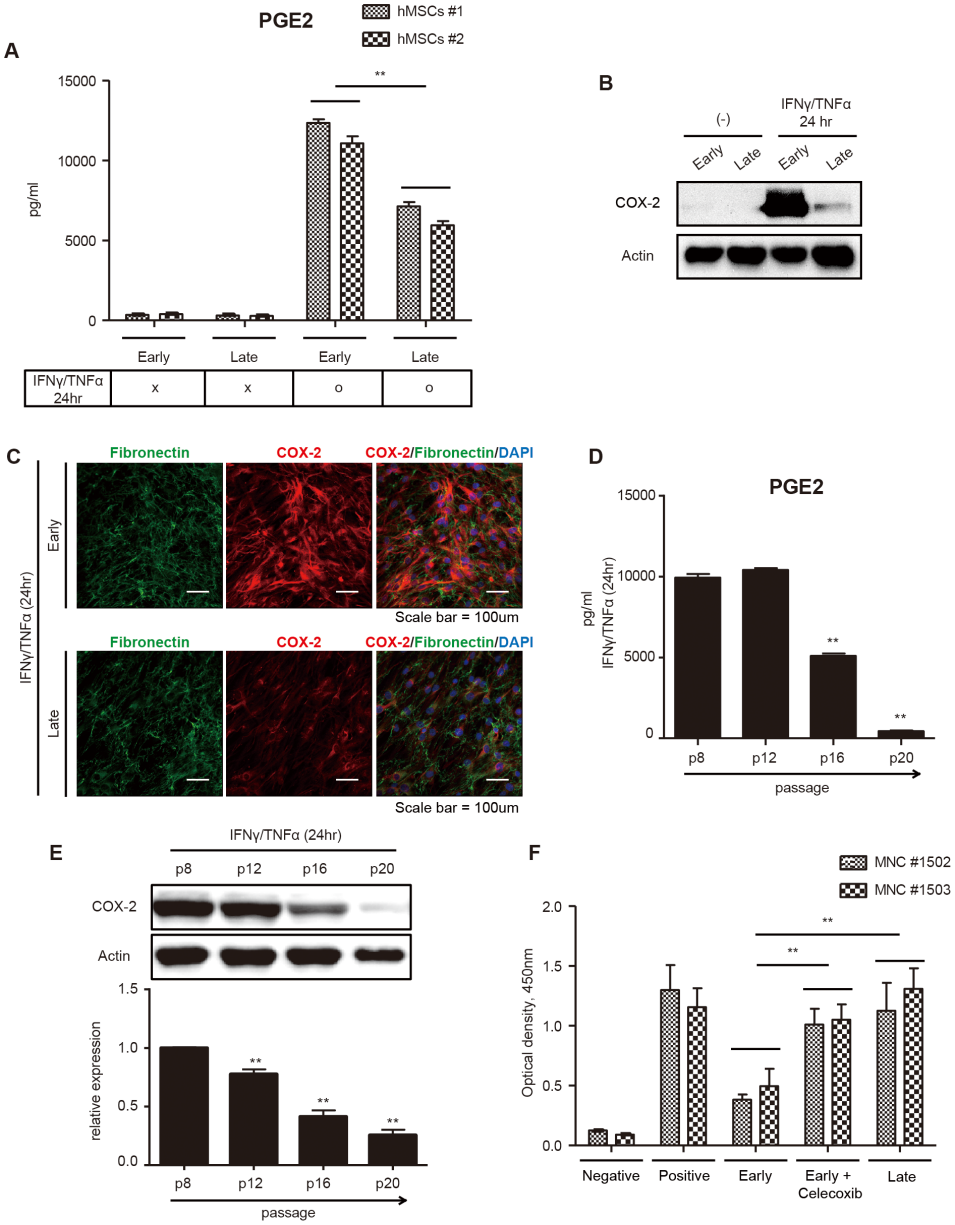
Declined immune-inhibitory effect of late-passage hMSCs is regulated by PGE2 and COX-2. (A–E) Early- and late-passage hMSCs were treated with or without IFN-γ and TNF-α for 24 hours. (A, D) The PGE_2_ concentration was measured from the culture supernatant by an ELISA after IFN-γ and TNF-α treatment for 24 hours. n = 3. (**P<0.01) (B, C, E) COX-2 expression was investigated using Western blot and immunocytochemistry after exposure to IFN-γ and TNF-α for 24 hours. GAPDH was used for normalization. n = 3. (**P<0.01) (F) Concanavalin A stimulated hMNCs were cultured alone or co-cultured with early-, late- passage and celecoxib-treated hMSCs at a 1∶10 ratio (MSC:MNC). The proliferation of hMNCs was measured by a BrdU assay after 72 hours. n = 3. (**P<0.01).

NO, PGE_2_, IDO and TGF-β1 have previously been identified as relevant mediators in the regulation of the immunomodulatory properties of MSCs [25]. To determine whether compromised immunosuppressive functions in hMSC aging are affected by these mediators, we measured the expression levels of each molecule from the culture media of early- and late-passage hMSCs. Because previous reports have shown evidence that the immunosuppressive functions of MSCs are elicited by proinflammatory cytokines [6], early- and late-passage hMSCs were primed with IFN-γ and TNF-α for 24 hours. There was no significant difference in the NO and TGF-β1 levels in the presence or absence of proinflammatory cytokines in both early- and late-passage hMSCs (Figure S2A and B). However, the PGE_2_ concentration was dramatically increased in the presence of proinflammatory cytokines. Interestingly, the PGE_2_ concentration in early-passage hMSCs was higher than in late-passage hMSCs (Figure 4A). We next investigated the expression levels of immunomodulatory molecules after proinflammatory cytokine activation using Western blot analysis. Consistent with the results from an ELISA assay, only the expression of COX-2, a key enzyme in production of PGE_2_, was significantly decreased, while the expression of p16^INK4A^, a senescence marker, was increased in late-passage hMSCs (Figure 4B and S2C). Furthermore, COX-2 expression was significantly down-regulated in late-passage hMSCs compared to early-passage hMSCs, whereas Fibronectin expression was not changed, which was confirmed by immunocytochemistry (Figure 4C). To identify whether the expression of PGE_2_ and COX-2 decreased following hMSC passages, we investigated the secretion level of PGE_2_ and the expression of the COX-2 protein in consecutive passages of hMSCs. The PGE_2_ concentration and COX-2 expression decreased gradually with the increasing hMSC passage number (Figure 4D and E). To determine the effect of COX-2/PGE_2_ on hMSC immunosuppressive functions, we observed the inhibitory effect of hMSCs on hMNC proliferation in the presence or absence of celecoxib, a selective COX-2 inhibitor. Celecoxib-treated, early-passage hMSCs exhibited significantly compromised immunosuppressive abilities (Figure 4F). Taken together, these results indicate that the immunosuppressive properties of hMSCs gradually decrease upon consecutive passages via down-regulation of COX-2/PGE_2_ expression.

### p38 MAP kinase is responsible for the reduced expression of COX-2

**Figure 5:**
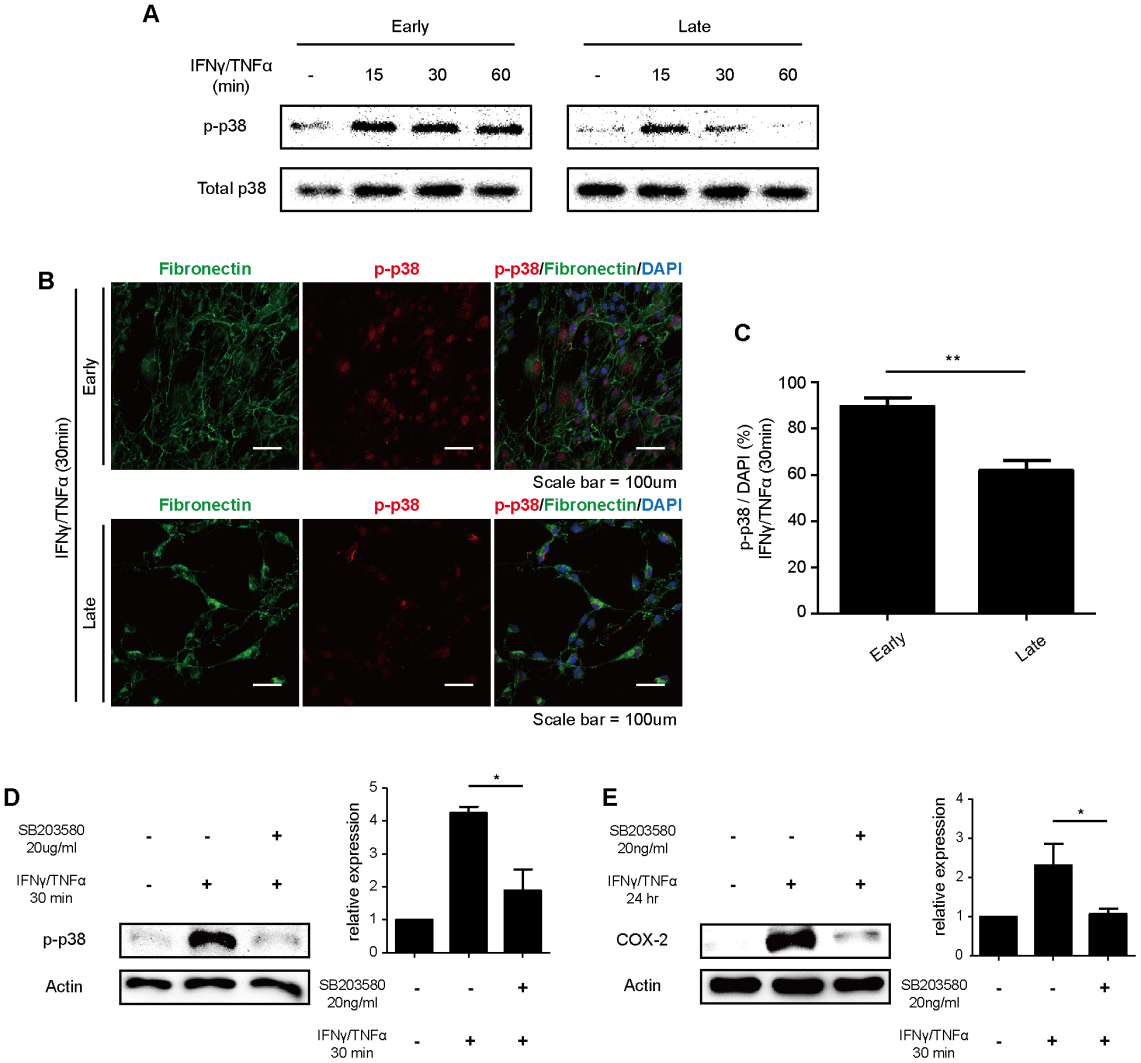
p38 MAP kinase is responsible for senescence-associated COX-2 expression. (A) Phosphorylation of p38 MAPK was investigated by Western blot analysis after treatment with IFN-γ and TNF-α, as indicated. (B, C) Expression of the phosphorylated form of p38 MAPK in early- and late-passage hMSCs was determined by immunocytochemistry after treatment with IFN-γ and TNF-α for 30 minutes. (D, E) After treating cells with SB203580, the expression levels of COX-2 and p-p38 were confirmed by Western blot analysis. Cells were treated with IFN-γ and TNF-α for 30 minutes to detect the expression of p-p38 and for 24 hours to detect COX-2 expression. n = 3. (*P<0.05).

p38 MAPK is one of the major intracellular signaling pathways activated by inflammatory stimuli, such as IFN-γ and TNF-α. It has been reported that p38 MAPK plays a significant role in activating the immune response by regulating PGE_2_/COX-2 synthesis [17], [26]. To determine whether the p38 MAPK pathway is activated upon hMSC aging, we examined the phosphorylation level of p38 MAPK. Phosphorylation of p38 MAPK was determined by Western blot analysis after the treatment of hMSCs with IFN-γ and TNF-α Early-passage hMSCs showed a higher level and duration of p38 MAPK activation without affecting the expression of IFN-γ and TNF-α receptors (Figure 5A and S3A). Our previous studies have demonstrated that COX-2/PGE_2_ expression is associated with the NOD2-RIP2 pathway, so we investigated the expression levels of NOD1 and NOD2 in early- and late-passage hMSCs. NOD1 and NOD2 expressions were not changed in early- and late-passage hMSCs (Figure S3B and C). Phosphorylation of p38 MAPK was also investigated immunocytochemically after exposure to IFN-γ and TNF-α and was found to decrease in late-passage hMSCs compared to early-passage hMSCs (Figure 5B and C). To confirm whether COX-2 expression was dependent on the phosphorylation of p38 MAPK, we investigated the expression of phosphorylated p38 MAPK and COX-2 after the treatment of hMSCs with SB203580, a p38 specific inhibitor. hMSCs were pretreated with SB203580 for 1 hour and were then stimulated with IFN-γ and TNF-α for 30 minutes to confirm the phosphorylation of p38 MAPK and for 24 hours for COX-2 expression. The expression of phosphorylated p38 MAPK and COX-2 was significantly decreased by treatment with SB203580 (Figure 5D and E). These results suggested that the impaired activation of p38 MAPK in late-passage hMSCs might lead to the down-regulation of COX-2/PGE_2_ expression.

## Discussion

The results presented here are the demonstration that replicative senescence in hMSCs impairs the secretion of soluble factors in response to the inflammatory milieu, thereby resulting in the loss of the immunoregulatory functions of hMSCs. Several studies have reported that cellular senescence affects the proliferation, multilineage differentiation and soluble factor production of MSCs [27]–[30]. However, none of these studies investigated whether cellular senescence affects their immunomodulatory ability. In this study, serial passaging by long-term culture significantly decreased the inhibitory effect of hMSCs on the mitogen-induced proliferation of hMNCs. This finding led us to confirm the in vivo anti-inflammatory effect of hMSCs using a chemically induced colitis model in which we previously verified the therapeutic effect of hMSCs [14]. Consistent with in vitro co-culture experiments, the protective effect of early-passage hMSCs against DSS-induced colitis in mice was completely abrogated when late-passage hMSCs were administered. Recently, Scruggs et al. [31] reported that human adipose-derived MSCs from old donors failed to ameliorate mouse experimental autoimmune encephalomyelitis, a model for human multiple sclerosis. Although this result correlates with our study in the respect that donor age or long-term culture affected the immunoregulatory ability of hMSCs, a further study may be required to elucidate the precise senescence-related characteristics of old donor-derived MSCs and long-term cultured, late-passage MSCs.

A previous study by Liu et al. [32] showed that the immunosuppressive properties of MSCs decreased with increasing passage. The main finding of this study was that although mouse MSCs sustained the secretion of TGF-β during long-term cultivation, the immunosuppressive ability of MSCs was diminished as their immunogenicity concomitantly increased. Similarly, in our study, the basal secretion level of soluble factors from hMSCs, including NO, TGF-β1 and PGE_2_, was not affected by cellular senescence. However, when hMSCs were treated with IFN-γ and TNF-α, prominent cytokines in type 1 helper T cell-mediated autoimmune diseases, such as inflammatory bowel disease, multiple sclerosis and type I diabetes, early-passage hMSCs produced a much higher level of PGE_2_ in response to cytokine treatment compared to late-passage hMSCs. In addition, the elevation in PGE_2_ production and COX-2 expression of hMSCs by cytokine treatment gradually diminished as passage increased. Furthermore, we showed that celecoxib-induced COX-2 inhibition in early-passage hMSCs diminished the suppressive effect of hMSCs on hMNC proliferation to the same extent observed in late-passage hMSCs. These results imply that PGE_2_ is a critical soluble factor through which hMSCs exert their suppressive effect on MNC and that the responsiveness of hMSCs against inflammatory cytokines might be the main reason for the loss of immunomodulatory functions in late-passage hMSCs, regardless of their immunogenicity. Liu et al. [32] also demonstrated that TGF-β from MSCs and IL-10 from MNCs were pivotal regulatory factors for an in vitro immune-inhibitory effect but not for an in vivo effect, indicating that factors other than TGF-β or IL-10 are involved in the physiological function of MSCs. Our previous study showed that hMSCs alleviated the DSS-induced colitis model through the production of PGE_2_ and that COX-2 inhibition led to the loss of this therapeutic effect [14]. Based on these findings, one can envision that hMSC-derived PGE_2_ may be the crucial factor for the regulation of both cellular and physiological inflammation and that impaired PGE_2_ production in senescent hMSCs upon an inflammatory trigger may result in the loss of their therapeutic effect against inflammatory diseases.

A recent study by Gu et al. [33] showed that human bone marrow-derived MSCs from systemic lupus erythematosus (SLE) patients were senescent and that the expression of p16^INK4A^, a major molecule that induces premature senescence, was significantly increased. Additionally, the inhibition of p16^INK4A^ expression reversed the senescent characteristics of MSCs via the activation of the extracellular signal regulated kinase (ERK) pathway, resulting in the up-regulation of TGF-β production and regulatory T cell induction. Therefore, in this study, we explored the key signaling pathway responsible for the senescence-mediated loss of responsiveness to inflammatory stimuli. Interestingly, phosphorylation of p38 MAPK upon stimulation with IFN-γ and TNF-α was decreased in late-passage hMSCs, whereas ERK signaling upon cytokine stimulation was not affected by cellular senescence (data not shown). It has been reported that NOD2 stimulation induces ERK and p38 activation mediated by RIP2 [34]. We also recently showed that NOD2 regulates the functions of hMSCs [35] and is required to suppress mononuclear cell proliferation by inducing production of PGE_2_ [14]. However, we found that expression of the *NOD2* gene was not significantly altered by serial *in vitro* passages, suggesting NOD2 signaling pathway is not implicated in decline of immunomodulatory property in late-passage hMSCs. Therefore, it is apparent that future work will require to elucidate the key signaling pathway responsible for the senescence-mediated decline of immunomodulatory property to restore the therapeutic function of immunosenescent hMSCs.

We conclude that consecutive cell divisions for the ex vivo expansion of hMSCs may alter their immunomodulatory properties. We show that PGE_2_ and COX-2, a key enzyme of PGE2 production, play positive roles in the immunomodulatory abilities of hMSCs. In addition, the immunomodulatory ability of hMSCs gradually declines with successive passages through a p38 MAP kinase-dependent change of the PGE_2_ and COX-2 levels. This study provides new insights into the mechanisms that regulate hMSC aging and may have implications for prolonging the effect of hMSCs in therapeutic applications.

## Materials and Methods

### Isolation and culture of hMSCs

Umbilical cord blood samples were obtained from the umbilical vein with the written informed consent of the mother and the approval of the Boramae Hospital Institutional Review Board (IRB) and the Seoul National University IRB (IRB no. 1109/001-006). The UCB samples were mixed with the HetaSep solution (StemCell Technologies, Vancouver, Canada) at a ratio of 5∶1 and were incubated at room temperature to deplete the erythrocytes. The supernatant was carefully collected, and the mononuclear cells were isolated using Ficoll density-gradient centrifugation at 1200 g for 20 minutes. The cells were washed twice with PBS. Cells were seeded at a density of 2×10^5^ to 2×10^6^ cells/cm^2^ on plates in growth medium consisting of D-medium (formula no. 78-5470EF, Gibco BRL, Grand Island, NY) containing EGM-2 SingleQuot and 10% fetal bovine serum (Gibco BRL). After 3 days, the non-adherent cells were removed. For long-term culture, the cells were seeded at a density of 4×10^5^ cells/10 cm plate and the cells were subcultured upon reaching ∼80–90% confluency.

### Flow cytometric analysis

hMSCs were triturated into single cells and labeled with monoclonal mouse anti-human fluorochrome-conjugated antibodies: CD29-PE, CD34-FITC, CD45-FITC, CD105-FITC, CD73-PE, and HLA-DR (BD Bioscience, San Jose, CA). The labeled cells were analyzed by flow cytometry using a FACS calibur (BD Biosciences).

### In vitro differentiation assay

The in vitro differentiation assay was performed as previously described [36]. For differentiation into osteoblasts and adipocytes, 1×10^5^ cells were plated in 6-well plates. After the cells reached 70–80% confluency, they were treated with an osteogenic differentiation induction medium (DMEM containing 10% FBS, 100 nM dexamethasone, 50 µM ascorbic acid 2-phosphate, and 10 mM β-glycerophosphate) or an adipogenic differentiation induction medium (DMEM supplemented with 10% FBS and 200 µM indomethacin, 1 µM dexamethasone, 0.5 mM isobutyl methylxanthine, and 0.5 µg/ml insulin) (all materials from Sigma-Aldrich, St. Louis, MO). The medium was changed every 3 days. After 2 weeks of induction, the cells were stained to confirm osteogenic or adipogenic differentiation. To confirm osteogenic differentiation, Alizarin Red S staining, which is specific for calcium, was performed to detect alkaline phosphate activity. Briefly, cells were rinsed with PBS and fixed with 70% ice-cold ethanol for 1 hour at 4°C. After 3 washes with distilled water, the cells were stained using 40 mM Alizarin Red S (Sigma-Aldrich) for 10 minutes at room temperature. To confirm the adipogenic differentiation, Oil Red O staining was conducted to detect fat droplets in differentiated cells. Briefly, cells were fixed with 10% formalin for 1 hour and were rinsed with 60% isopropanol before incubation in fresh diluted Oil Red O for 10 minutes. For chondrogenic differentiation, 5×10^5^ cells were placed in a 15-mL polypropylene tube and maintained with 1 mL of chondrocyte differentiation medium (Lonza, Wakersville, MD) for 3 weeks. The round pellets were embedded in paraffin and cut into 3-µm sections. The sections were stained with toluidine blue to detect glycosaminoglycans.

### Isolation and culture of hUCB-derived MNCs

Human mononuclear cells were isolated from UCB samples. The UCB samples were mixed with the HetaSep solution (StemCell Technologies) at a ratio of 5∶1 and were then incubated at room temperature to deplete erythrocyte counts. The supernatant was carefully collected and mononuclear cells were obtained using Ficoll density-gradient centrifugation at 2,500 rpm for 20 minutes. The cells were washed twice in PBS and seeded in growth media consisted of RPMI 1640 (Gibco BRL) containing 10% fetal bovine serum.

### Senescence-associated betagalactosidase (SA β-gal) staining

SA β-gal staining was performed as previously described [21]. The hMSCs were seeded on 6-well plates at a density of 1×10^5^ cells/well for late-passage cells and 5×10^4^ cells/well for early-passage cells. The cells were incubated for 3 days until they reached the appropriate confluency. The cells were then washed twice with PBS and fixed with 0.5% glutaraldehyde in PBS (pH 7.2) for 5 minutes at room temperature. The cells were then washed with PBS containing MgCl_2_ (pH 7.2, 1 mM MgCl_2_) and stained with X-gal solution [1 mg/ml X-gal, 0.12 mM K_3_Fe(CN)_6_, 1 mM MgCl_2_ in PBS, pH 6.0] overnight at 37°C. The cells were washed twice with PBS and the images were captured using a microscope (IX70, Olympus, Japan).

### Cumulative population doubling level

hMSCs were maintained in a medium containing 10% FBS and were subcultured every 3 day. The proliferation potential of early-passage and late-passage hMSCs was determined by calculating the cumulative population doubling level in continual subculture and growth from a known number of cells. At each subculture, the CPDL was calculated from the cell count using the equation: ln(N_f_/N_i_)/ln2, where N_i_ and N_f_ are the initial and final cell count numbers, respectively, and ln is the natural log. A total number of 5×10^4^ cells were initially plated in a 6-well culture plate (Nunc, Rochester, NY) and were counted at each subculture. Calculated CPDL rates were added serially and represented as a broken line graph.

### Proliferation assay

hMSCs were treated with 25 µg/ml of mitomycin C (Mitomycin C from *Streptomyces caespitosus*) at 37°C for 1 hour. After washing twice in PBS, the cells were seeded in 96-well plates at 1×10^4^/well and were incubated for 24 hours. hMNCs were prepared as previously described, treated with Concanavalin A (Con A from *Canavalia ensiformis*) in RPMI medium and added cultured at 1×10^5^ cells/well. After 3 days of co-culture, MNC proliferation was determined using a cell proliferation ELISA kit, BrdU kit (Roche, Indianapolis, IN). For the activation of T cells, CD3/CD28 beads (Invitrogen Life Technologies, Carlsbad, CA) were added according to manufacturer's instruction followed by additional treatment of recombinant human interleukin-2 (30 U/mL, R&D systems, Minneapolis, MN). Changes of absorbance (optical density, OD), as the level of BrdU incorporation, were measured spectrophotometrically at 450 nm in a microplate reader.

### Mice and Ethical Statement

C57BL/6J mice (male; aged 8–10 weeks; 18–25 g) were obtained from Jackson Laboratory (Bar Harbor, ME). Mice were group-housed in the animal facility of Seoul National University. All experiments were conducted at the Seoul National University. All of the experiments were approved by and followed the regulations of the Institute of Laboratory Animals Resources (SNU-131209-1 Seoul National University). The experimental protocol was approved by the Seoul National University Institutional Animal Care and Use Committee (SNU IACUC). Institutional guidelines for animal care and use were followed throughout the experiments. Mice were monitored until they had reached criteria for humane endpoints. Mice losing above 30% of their original body weight or showing signs of distress (unphysiological bodily posture, shaggy fur, and breathlessness/panting etc) were sacrificed using CO_2_ asphyxiation. At the designated time points for tissue harvesting, mice were sacrificed by CO_2_-asphyxiation. Every effort was made to minimize suffering.

### Colitis induction

This study was performed in strict accordance with the recommendations in the Guide for the Care and Use of Laboratory Animals of the National Institutes of Health. Colitis was induced by administration of dextran sulfate sodium (DSS, MP Biochemicals, Solon, OH) in drinking water as previously reported [37]. Briefly, colitis was induced in mice from specific pathogen-free (SPF) facilities by the administration of 3% (w/v) DSS in drinking water for 7 days followed by normal drinking water until the end of the experiment. Early- and late-passage hUCB-MSCs resuspended in PBS (2×10^6^ cells in 200 µl) were injected intraperitoneally into mice 1 day after the administration of DSS. Colitis-induced mice were randomly assigned to the following groups in experiment. The mice were checked each day for morbidity (n = 12 for negative control mice, n = 12 for positive control mice, n = 12 for early-passage hMSC injected mice and n = 12 for late-passage hMSC injected mice) and body weight was recorded over 14 days at a specific time of a day (n = 6 for negative control mice, n = 18 for positive control mice, n = 15 for early-passage hMSC injected mice and n = 15 for late-passage hMSC injected mice). At day 9, colitis severity was measured by evaluating the disease activity index through the scoring of weight loss (0∼4), stool consistency (0∼4), bleeding (0∼4), coat roughness (0∼4), mouse activity (0∼2), and bedding contamination by stool and blood (0∼2). At the peak of the disease (day 10), the mice were sacrificed by CO_2_-asphyxiation and their colon length and weight were measured (n = 7 for negative control mice, n = 15 for positive control mice, n = 16 for early-passage hMSC injected mice and n = 16 for late-passage hMSC injected mice). Histopathological evaluation was performed with the tissue acquired.

### Histopathologic Evaluation

Colon samples were collected, fixed in 10% formalin, subjected to consecutive steps of alcohol–xylene changes, and embedded in paraffin. Sections that were 5-mm thick were prepared and stained with H&E. Scattered infiltration of inflammatory cells in the lamina propria and submucosa (0∼4) and intestinal damage based on destruction of entire epithelium and severe submucosal edema (0∼4) were graded blindly.

### Cytokine production

Cells were treated with recombinant human IFN-γ and TNF-α (Peprotech. Rocky Hill, NJ) for 24 hours, and PGE_2_ production was determined from the culture supernatant using a commercial ELISA kit (R&D Systems). For the NO measurement, hMSCs were treated with IFN-γ and TNF-α for 24 hours, and the concentration was measured using an ELISA kit (Promega, Madison, WI). TGF-β1 production was measured from the MSC culture supernatant after treatment of IFN-γ and TNF-α for 24 hours using a commercial ELISA kit (eBioscience, San Diego, CA).

### Western blot analysis

After treatment with recombinant human IFN-γ and TNF-α (Peprotech) for 24 hours or 15 to 60 minutes, whole cell protein lysates were extracted in a solution containing 1% Triton X-100, 20 mM Tris HCL (pH 8), 137 mM NaCl, 10% glycerol and 2 mM EDTA (Sigma-Aldrich), and the protein concentrations were determined using a DC assay kit (Bio-Rad, Hercules, CA). The proteins were separated by 10% sodium dodecyl sulfate polyacrylamide gel electrophoresis (SDS-PAGE), transferred to nitrocellulose membranes at 100 V and 350 mA for 2 hours and probed with primary antibodies. The primary antibodies used to detect each protein were as follows: COX-2 (polyclonal, Abcam, Cambridge, MA, 1∶1000), p-p38 (polyclonal, Cell Signaling, Danvers, MA, 1∶1000), p38 (monoclonal, Abcam, 1∶1000), IFNGR1 (polyclonal, Abcam, 1∶1000), TNFR (monoclonal, Millipore, Billerica, MA 1∶1000), p16^INK4A^ (monoclonal, Abcam, 1∶1000), Glyceraldehyde-3-Phosphate Dehydrogenase (monoclonal, Millipore, 1∶3000), and β-actin (monoclonal, Cell-signaling, 1∶5000). The secondary antibodies were used according to the manufacturer's specifications and binding was detected using an enhanced chemiluminescence (ECL) detection kit (Amersham, Piscataway, NJ) according to the manufacturer's instructions.

### Immunocytochemistry

Cells were fixed in 4% paraformaldehyde for 10 min at room temperature after treatment with recombinant human IFN-γ and TNF-α (Peprotech) for 30 min or 24 hours. The cells were permeabilized by exposure to 0.5% Triton X-100 in PBS for 10 min and were then blocked for 2 h with 10% normal goat serum (Zymed, San Francisco, CA) at room temperature. The cells were then stained with antibodies against COX-2 (polyclonal, Abcam, 1∶200) or p-p38 (polyclonal, Cell Signaling, 1∶200), followed by incubation for 1 hour with an Alexa-Fluor-488- or Alexa-Fluor-594-labeled secondary antibody (1∶1000; Molecular Probes, Oregon). The nuclei were stained with Hoechst 33258 (1 µg/ml for 10 minutes), and the images were captured using a confocal microscope (Eclipse TE200, Nikon, Japan).

### Reverse transcription polymerase chain reaction (RT-PCR) and real-time PCR

The total cellular RNA was extracted from cells using the TRIzol reagent (Invitrogen, Carlsbad, CA) according to the manufacturer's instructions. Purified RNA was transcribed into complementary DNA using Super-script III First-Strand Synthesis System (Invitrogen) followed by quantitative PCR using the SYBR Green PCR Master Mix (Applied Biosystems, Foster City, CA) with each primer. According to the manufacturer's intructions, AB 7500 Software Version 2.0.5 (Applied Biosystems) was used to quantify gene expression. Each gene expression level was normalized with GAPDH as housekeeping controls. At least three independent analyses were carried out for each gene. The following primers were used: for NOD1 forward, 5′-CCACTTCACAGCTGGAGACA-3′ and reverse, 5′- TGAGTGGAAGCAGCATTTTG-3′; for NOD2 forward, 5′-CCTGAATGTTGGGCACCTCA-3′ and reverse, 5′-CTTGCAGACACCAAGGCAAG-3′; for GAPDH forward, 5′-TGATGACATCAAGAAGGTGGTG-3′ and reverse, 5′- ACCCTGTTGCTGTAGCCAAAT-3′.

### Statistical analysis

All of the experiments were conducted at least three times (n = 3) and the results are expressed as the mean ± SD. All of the statistical comparisons were made by one-way analysis of variance followed by a Bonferroni post hoc test for multigroup comparisons using GraphPad Prism software (version 5.01; GraphPad Software, San Diego, CA). Statistical significance designated is indicated in the Figure Legends.

## Supporting Information

Figure S1 **Early-passage hMSCs and late-passage hMSCs show different inhibition rates of MNC proliferation.** Inhibition rates of hMNC proliferation in the presence of early- and late-passage hMSCs were calculated. Ten tests were performed. (*P<0.05).
(TIF)

Figure S2 **Replicative senescence results in no change in NO and TGF-β1 secretion.** (A) NO secretion levels were investigated in early- and late-passage hMSCs after treatment with IFN-γ and TNF-α. (B) Secretion of TGF-β1 was measured in early- and late-passage hMSC culture media after the concomitant addition of IFN-γ and TNF-α. (C) Expression of IDO, Galectin 3 and inducible Nitric Oxide Synthase (iNOS) showed no difference in early- and late-passage hMSCs.
(TIF)

Figure S3 **Expression of receptors shows no difference between early- and late-passage hMSCs.** (A) After exposure to IFN-γ and TNF-α for 30 minutes, Western blot analysis was performed to confirm the expression levels of each cytokine receptor. (B, C) Early- and late- passage hMSCs were analyzed for mRNA expression of NOD1 (B) and NOD2 (C) using real-time RT-PCR. Error bars denote standard deviation of triplicate reactions.
(TIF)

## References

[1] Chamberlain G, Fox J, Ashton B, Middleton J (2007) Concise review: mesenchymal stem cells: their phenotype, differentiation capacity, immunological features, and potential for homing. Stem Cells 25: 2739–2749. 17656645 10.1634/stemcells.2007-0197

[2] Pittenger MF, Mackay AM, Beck SC, Jaiswal RK, Douglas R, et al (1999) Multilineage potential of adult human mesenchymal stem cells. Science 284: 143–147. 10102814 10.1126/science.284.5411.143

[3] Uccelli A, Moretta L, Pistoia V (2008) Mesenchymal stem cells in health and disease. Nat Rev Immunol 8: 726–736. 19172693 10.1038/nri2395

[4] Karp JM, Leng Teo GS (2009) Mesenchymal stem cell homing: the devil is in the details. Cell Stem Cell 4: 206–216. 19265660 10.1016/j.stem.2009.02.001

[5] Shi Y, Hu G, Su J, Li W, Chen Q, et al (2010) Mesenchymal stem cells: a new strategy for immunosuppression and tissue repair. Cell Res 20: 510–518. 20368733 10.1038/cr.2010.44

[6] Ren G, Zhang L, Zhao X, Xu G, Zhang Y, et al (2008) Mesenchymal stem cell-mediated immunosuppression occurs via concerted action of chemokines and nitric oxide. Cell Stem Cell 2: 141–150. 18371435 10.1016/j.stem.2007.11.014

[7] Ren G, Su J, Zhang L, Zhao X, Ling W, et al (2009) Species variation in the mechanisms of mesenchymal stem cell-mediated immunosuppression. Stem Cells 27: 1954–1962. 19544427 10.1002/stem.118

[8] Meisel R, Zibert A, Laryea M, Gobel U, Daubener W, et al (2004) Human bone marrow stromal cells inhibit allogeneic T-cell responses by indoleamine 2,3-dioxygenase-mediated tryptophan degradation. Blood 103: 4619–4621. 15001472 10.1182/blood-2003-11-3909

[9] Nemeth K, Leelahavanichkul A, Yuen PS, Mayer B, Parmelee A, et al (2009) Bone marrow stromal cells attenuate sepsis via prostaglandin E(2)-dependent reprogramming of host macrophages to increase their interleukin-10 production. Nat Med 15: 42–49. 19098906 10.1038/nm.1905 PMC2706487

[10] Spaggiari GM, Abdelrazik H, Becchetti F, Moretta L (2009) MSCs inhibit monocyte-derived DC maturation and function by selectively interfering with the generation of immature DCs: central role of MSC-derived prostaglandin E2. Blood 113: 6576–6583. 19398717 10.1182/blood-2009-02-203943

[11] Spaggiari GM, Capobianco A, Abdelrazik H, Becchetti F, Mingari MC, et al (2008) Mesenchymal stem cells inhibit natural killer-cell proliferation, cytotoxicity, and cytokine production: role of indoleamine 2,3-dioxygenase and prostaglandin E2. Blood 111: 1327–1333. 17951526 10.1182/blood-2007-02-074997

[12] Aggarwal S, Pittenger MF (2005) Human mesenchymal stem cells modulate allogeneic immune cell responses. Blood 105: 1815–1822. 15494428 10.1182/blood-2004-04-1559

[13] Lin CS, Lin G, Lue TF (2012) Allogeneic and xenogeneic transplantation of adipose-derived stem cells in immunocompetent recipients without immunosuppressants. Stem Cells Dev 21: 2770–2778. 22621212 10.1089/scd.2012.0176 PMC3806387

[14] Kim HS, Shin TH, Lee BC, Yu KR, Seo Y, et al (2013) Human umbilical cord blood mesenchymal stem cells reduce colitis in mice by activating NOD2 signaling to COX2. Gastroenterology 145: 1392–1403 e1391–1398 23973922 10.1053/j.gastro.2013.08.033

[15] Greenhough A, Smartt HJ, Moore AE, Roberts HR, Williams AC, et al (2009) The COX-2/PGE2 pathway: key roles in the hallmarks of cancer and adaptation to the tumour microenvironment. Carcinogenesis 30: 377–386. 19136477 10.1093/carcin/bgp014

[16] Saklatvala J (2004) The p38 MAP kinase pathway as a therapeutic target in inflammatory disease. Curr Opin Pharmacol 4: 372–377. 15251131 10.1016/j.coph.2004.03.009

[17] Dean JL, Brook M, Clark AR, Saklatvala J (1999) p38 mitogen-activated protein kinase regulates cyclooxygenase-2 mRNA stability and transcription in lipopolysaccharide-treated human monocytes. J Biol Chem 274: 264–269. 9867839 10.1074/jbc.274.1.264

[18] Banfi A, Bianchi G, Notaro R, Luzzatto L, Cancedda R, et al (2002) Replicative aging and gene expression in long-term cultures of human bone marrow stromal cells. Tissue Eng 8: 901–910. 12542936 10.1089/107632702320934001

[19] Wagner W, Horn P, Castoldi M, Diehlmann A, Bork S, et al (2008) Replicative senescence of mesenchymal stem cells: a continuous and organized process. PLoS One 3: e2213. 18493317 10.1371/journal.pone.0002213 PMC2374903

[20] Jung JW, Lee S, Seo MS, Park SB, Kurtz A, et al (2010) Histone deacetylase controls adult stem cell aging by balancing the expression of polycomb genes and jumonji domain containing 3. Cell Mol Life Sci 67: 1165–1176. 20049504 10.1007/s00018-009-0242-9 PMC2835723

[21] Yu KR, Lee S, Jung JW, Hong IS, Kim HS, et al (2013) MicroRNA-141-3p plays a role in human mesenchymal stem cell aging by directly targeting ZMPSTE24. J Cell Sci 126: 5422–5431. 24101728 10.1242/jcs.133314

[22] Yu KR, Kang KS (2013) Aging-related genes in mesenchymal stem cells: a mini-review. Gerontology 59: 557–563. 23970150 10.1159/000353857

[23] Cooper HS, Murthy SN, Shah RS, Sedergran DJ (1993) Clinicopathologic study of dextran sulfate sodium experimental murine colitis. Lab Invest 69: 238–249. 8350599

[24] Andre C, Descos L, Landais P, Fermanian J (1981) Assessment of appropriate laboratory measurements to supplement the Crohn's disease activity index. Gut 22: 571–574. 6973509 10.1136/gut.22.7.571 PMC1419331

[25] Shi Y, Su J, Roberts AI, Shou P, Rabson AB, et al (2012) How mesenchymal stem cells interact with tissue immune responses. Trends Immunol 33: 136–143. 22227317 10.1016/j.it.2011.11.004 PMC3412175

[26] Chen C, Chen YH, Lin WW (1999) Involvement of p38 mitogen-activated protein kinase in lipopolysaccharide-induced iNOS and COX-2 expression in J774 macrophages. Immunology 97: 124–129. 10447723 10.1046/j.1365-2567.1999.00747.x PMC2326802

[27] Crisostomo PR, Wang M, Wairiuko GM, Morrell ED, Terrell AM, et al (2006) High passage number of stem cells adversely affects stem cell activation and myocardial protection. Shock 26: 575–580. 17117132 10.1097/01.shk.0000235087.45798.93

[28] Gruber HE, Somayaji S, Riley F, Hoelscher GL, Norton HJ, et al (2012) Human adipose-derived mesenchymal stem cells: serial passaging, doubling time and cell senescence. Biotech Histochem 87: 303–311. 22250760 10.3109/10520295.2011.649785

[29] Lee S, Park JR, Seo MS, Roh KH, Park SB, et al (2009) Histone deacetylase inhibitors decrease proliferation potential and multilineage differentiation capability of human mesenchymal stem cells. Cell Prolif 42: 711–720. 19689470 10.1111/j.1365-2184.2009.00633.x PMC6496408

[30] Zaim M, Karaman S, Cetin G, Isik S (2012) Donor age and long-term culture affect differentiation and proliferation of human bone marrow mesenchymal stem cells. Ann Hematol 91: 1175–1186. 22395436 10.1007/s00277-012-1438-x

[31] Scruggs BA, Semon JA, Zhang X, Zhang S, Bowles AC, et al (2013) Age of the donor reduces the ability of human adipose-derived stem cells to alleviate symptoms in the experimental autoimmune encephalomyelitis mouse model. Stem Cells Transl Med 2: 797–807. 24018793 10.5966/sctm.2013-0026 PMC3785264

[32] Liu H, Lu K, MacAry PA, Wong KL, Heng A, et al (2012) Soluble molecules are key in maintaining the immunomodulatory activity of murine mesenchymal stromal cells. J Cell Sci 125: 200–208. 22250196 10.1242/jcs.093070

[33] Gu Z, Cao X, Jiang J, Li L, Da Z, et al (2012) Upregulation of p16INK4A promotes cellular senescence of bone marrow-derived mesenchymal stem cells from systemic lupus erythematosus patients. Cell Signal 24: 2307–2314. 22820504 10.1016/j.cellsig.2012.07.012

[34] Yang Y, Yin C, Pandey A, Abbott D, Sassetti C, et al (2007) NOD2 pathway activation by MDP or Mycobacterium tuberculosis infection involves the stable polyubiquitination of Rip2. J Biol Chem 282: 36223–36229. 17947236 10.1074/jbc.M703079200

[35] Kim HS, Shin TH, Yang SR, Seo MS, Kim DJ, et al (2010) Implication of NOD1 and NOD2 for the differentiation of multipotent mesenchymal stem cells derived from human umbilical cord blood. PLoS One 5: e15369. 21042538 10.1371/journal.pone.0015369 PMC2962653

[36] Yu KR, Yang SR, Jung JW, Kim H, Ko K, et al (2012) CD49f enhances multipotency and maintains stemness through the direct regulation of OCT4 and SOX2. Stem Cells 30: 876–887. 22311737 10.1002/stem.1052

[37] Okayasu I, Hatakeyama S, Yamada M, Ohkusa T, Inagaki Y, et al (1990) A novel method in the induction of reliable experimental acute and chronic ulcerative colitis in mice. Gastroenterology 98: 694–702. 1688816 10.1016/0016-5085(90)90290-h

