# Correction: A p38 MAPK-Mediated Alteration of COX-2/PGE_2_ Regulates Immunomodulatory Properties in Human Mesenchymal Stem Cell Aging

**DOI:** 10.1371/journal.pone.0112030

**Published:** 2014-10-22

**Authors:** The PLOS ONE Staff

There are errors in the author affiliations. The affiliations should appear as shown here:

Kyung-Rok Yu^1,2^, Jin Young Lee^1,2^, Hyung-Sik Kim^1,3^, In-Sun Hong^4,5^, Soon Won Choi^1,2^, Yoojin Seo^1,2^, Insung Kang^1,2^, Jae-Jun Kim^1,2^, Byung-Chul Lee^1,2^, SeungHee Lee^1,2,3^, Andreas Kurtz^1,2,6^, Kwang-Won Seo^1,2,3^ and Kyung-Sun Kang^1,2,^

^1^ Adult Stem Cell Research Center, College of Veterinary Medicine, Seoul National University, Seoul 151-742, South Korea

^2^ Research Institute for Veterinary Medicine, College of Veterinary Medicine, Seoul National University, Seoul 151-742, Korea

^3^ Institute for Stem Cell and Regenerative Medicine in Kangstem Biotech, Biotechnology Incubating Center, Seoul National University, Seoul 151-742, Korea

^4^ Department of Molecular Medicine, Gachon University, Incheon, Republic of Korea

^5^ Lee Gil Ya Cancer and Diabetes Institute, Incheon, Republic of Korea

^6^ Berlin-Brandenburg Center for Regenerative Therapies, Berlin, Germany
