# Supplementary figures and images for: A p38 MAPK-Mediated Alteration of COX-2/PGE2 Regulates Immunomodulatory Properties in Human Mesenchymal Stem Cell Aging
Source: PLoS One. 2014 Aug 4;9(8):e102426. doi: 10.1371/journal.pone.0102426 (PMC4121064; doi:10.1371/journal.pone.0102426)

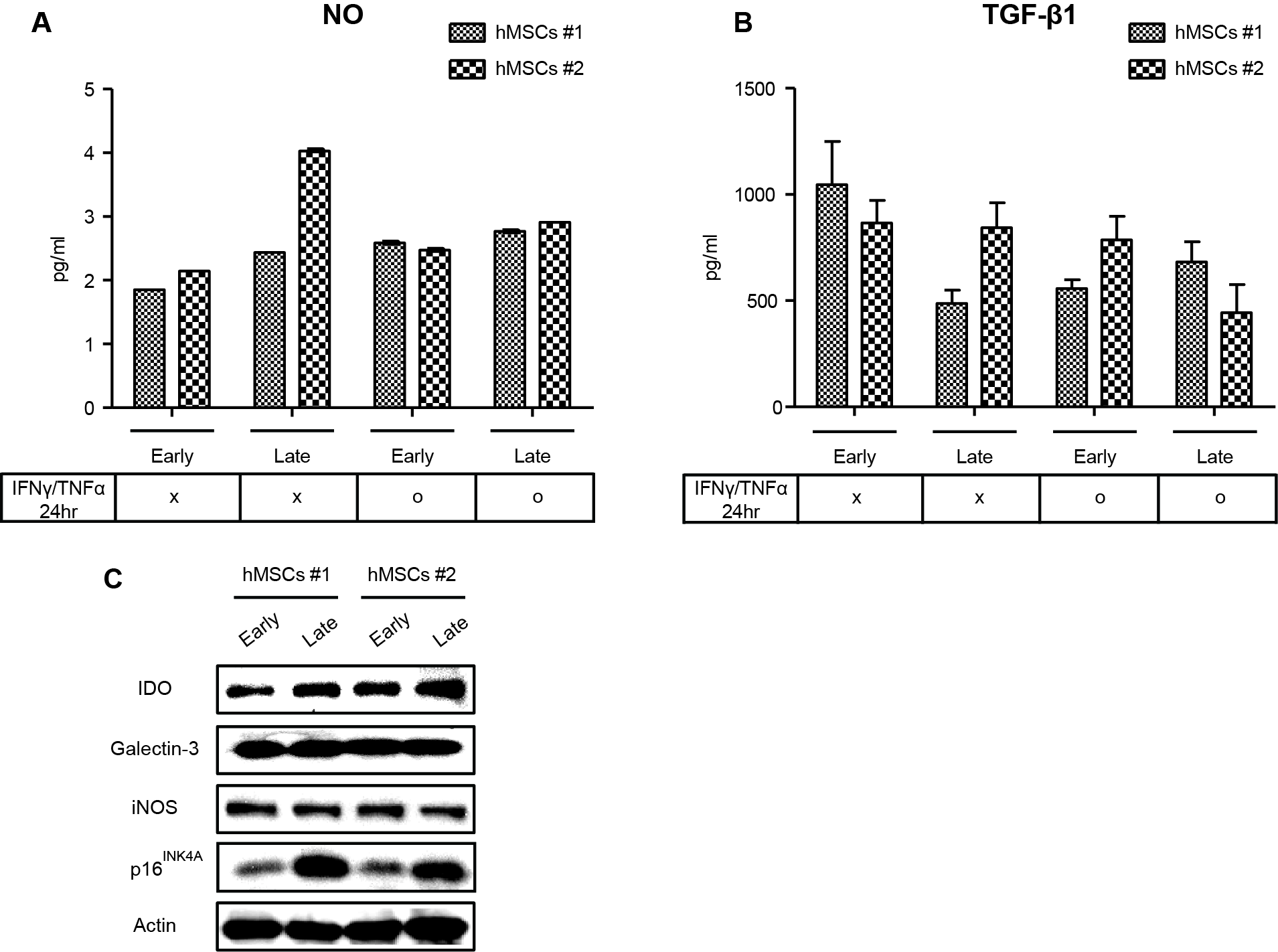

Supplement: Figure S2 — Replicative senescence results in no change in NO and TGF-β1 secretion. (A) NO secretion levels were investigated in early- and late-passage hMSCs after treatment with IFN-γ and TNF-α. (B) Secretion of TGF-β1 was measured in early- and late-passage hMSC culture media after the concomitant addition of IFN-γ and TNF-α. (C) Expression of IDO, Galectin 3 and inducible Nitric Oxide Synthase (iNOS) showed no difference in early- and late-passage hMSCs. (TIF) [file pone.0102426.s002.tif]

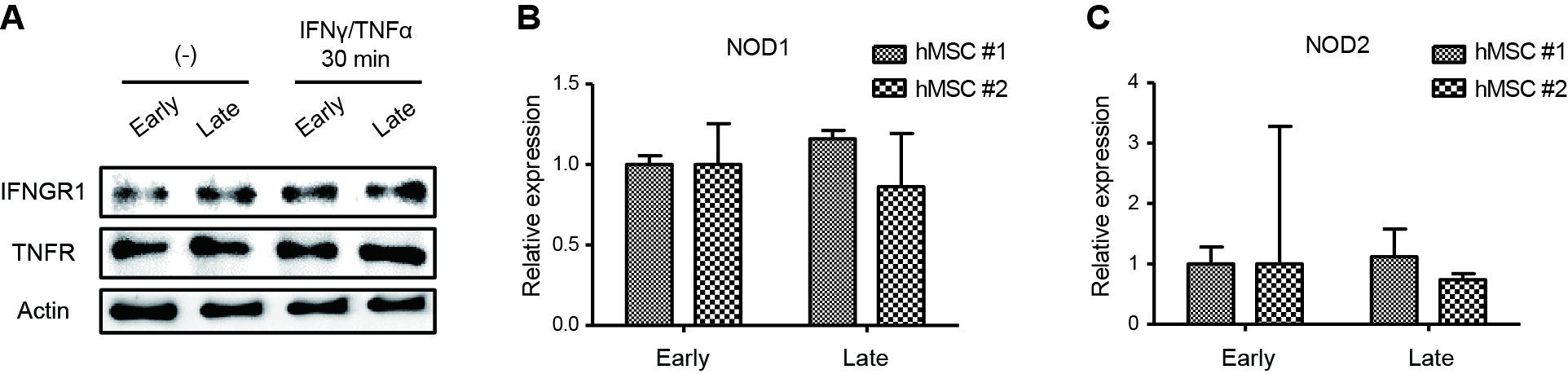

Supplement: Figure S3 — Expression of receptors shows no difference between early- and late-passage hMSCs. (A) After exposure to IFN-γ and TNF-α for 30 minutes, Western blot analysis was performed to confirm the expression levels of each cytokine receptor. (B, C) Early- and late- passage hMSCs were analyzed for mRNA expression of NOD1 (B) and NOD2 (C) using real-time RT-PCR. Error bars denote standard deviation of triplicate reactions. (TIF) [file pone.0102426.s003.tif]
